# Integrated Change Detection and Classification in Urban Areas Based on Airborne Laser Scanning Point Clouds

**DOI:** 10.3390/s18020448

**Published:** 2018-02-03

**Authors:** Thi Huong Giang Tran, Camillo Ressl, Norbert Pfeifer

**Affiliations:** 1 Department of Geodesy and Geoinformation, Technische Universität Wien, Gußhausstraße 27-29, 1040 Vienna, Austria; camillo.ressl@geo.tuwien.ac.at (C.R.); norbert.pfeifer@geo.tuwien.ac.at (N.P.); 2 Faculty of Geomatics and Land Administration, Hanoi University of Mining and Geology, Hanoi 10000, Vietnam

**Keywords:** LiDAR, change classification, machine learning

## Abstract

This paper suggests a new approach for change detection (CD) in 3D point clouds. It combines classification and CD in one step using machine learning. The point cloud data of both epochs are merged for computing features of four types: features describing the point distribution, a feature relating to relative terrain elevation, features specific for the multi-target capability of laser scanning, and features combining the point clouds of both epochs to identify the change. All these features are merged in the points and then training samples are acquired to create the model for supervised classification, which is then applied to the whole study area. The final results reach an overall accuracy of over 90% for both epochs of eight classes: lost tree, new tree, lost building, new building, changed ground, unchanged building, unchanged tree, and unchanged ground.

## 1. Introduction

Change detection plays an important role in keeping topographic databases up-to-date, in monitoring, and in planning [1]. One major data source for documenting landscape change are 2D satellite images, especially in large-scale problems as urbanization, forest monitoring, earthquake hazard and risk assessment, etc. [2,3,4,5,6,7]. For these tasks, many studies used low-to-medium resolution images [8,9,10,11,12], although high resolution images were also employed for change detection at a higher level [13,14,15]. However, high resolution 2D-based change detection has several limitations such as higher spectral variability [6], perspective distortion [16,17], and lack of volumetric information [18,19]. These limitations complicate 2D-based change detection. With three dimension (3D) geometric information, 3D change detection is not influenced by illumination, perspective distortion and illumination variations as 2D change detection [20]. The third dimension as a supplementary data source (height, full 3D information, or depth) and the achievable outcome (height differences, volumetric change) expand the scope of CD applications [20] in 3D city model updating [21,22], 3D structure and construction monitoring [23,24], 3D object tracking [25,26], tree growth monitoring and biomass estimation [27,28], and landslide surveillance [29].

An established source of detailed and accurate 3D information is airborne LiDAR (light detection and ranging), which provides a point cloud, and is applied in various fields [30,31,32,33]. Therefore, airborne LiDAR is creating new possibilities for 3D change detection, especially in urban areas where complex 3D situations prevail [34].

Many approaches suggested in the literature demonstrate the high potential of LiDAR point clouds for change detection (see Section 2). Most studies apply two steps: first, detect the change; and, second, classify the change; alternatively, first, classify objects for both periods; and, second, detect changes between the classifications. Both approaches, consequently, will be influenced by sequence error, i.e., the accuracy of classified changes depends on the change detection method and the classification method. Furthermore, most of those studies focus only on one class (typically either building or trees). However, a change typically does not happen in a single class only, but leads to changes in multiple classes. We are therefore suggesting to investigate the possibilities of performing change detection and classification of all the main classes (building, tree, and ground) simultaneously in one step.

In this paper, we suggest a new approach in change detection (CD). It combines classification and CD in one step. Additionally, it builds on the point cloud, which is a common data source for high resolution geoinformation from laser scanning and image matching alike. It exploits the power of machine learning [35]. Given two raw point clouds of different epochs, sampled training data is required only once. The method provides a separation whether there is a change or no change at the location of the point as well as individual class information for each point. The method is presented for one site, and its properties are discussed.

## 2. Related Work

We suggest to classify change detection approaches using ALS data in urban areas into two methods: “post-classification” CD and “pre-classification” CD. In the first category, post-classification, the urban objects are first classified into specific classes and then changes are detected in the classifications. In the second category, pre-classification, the differences between two datasets are detected first and then the change types are classified later. An overview of published approaches is shown in Table 1. The description will only focus on those articles, in which a specifically new aspect was added to the overall approach.

In post-classification CD, ALS data can either be combined with other datasets from a different epoch, or two different epochs of airborne LiDAR data are used. The former is a common method, investigated in many studies, incorporating the advantages of the LiDAR height data with either images [36,37,38] or existing maps for updating information [42,43,44]. Malpica et al. [39] proposed an approach that employed ALS data and satellite imagery for updating buildings of a geospatial vector database. LiDAR data were used to derive the height above the terrain, which was associated with spectral information and became the input for a support vector machine (SVM) classification. This method proved useful for tall buildings, yet small houses and low buildings surrounded by trees were not well-detected. Teo and Shih [47] suggested a CD method, in which the change in building types were obtained by handling multi-temporal interpolated LiDAR data. Recently, Matikanen et al. [40,41] demonstrated the potential of multispectral airborne laser in automated classification and change detection. Land cover classification was derived from multispectral ALS data using a random forest classifier. Afterwards, building changes were detected by combination of the land cover classification results with a digital surface model (DSM) and building vectors from a previous epoch. Road changes were detected by comparing road classes from the classification results with the road centerline vectors. The approaches mentioned above enable detecting changes in 2.5D (DSMs) or only in 2D (Maps), both of which may cause loss of information under trees. In contrast, two ALS data epochs facilitate overcoming this issue. Choi et al. [45] based change detection on a DSM subtraction between two ALS epochs to detect change areas. The points within the detected areas were then organized into surface patches, which were subsequently classified into ground, vegetation, and building. The type of the change was determined based on the classes and properties of each patch. Xu et al. [46] detected the changes in buildings from commercial (acquired in the years 2008 and 2010), and residential area (2010 and 2012) by two epoch ALS data. Their “scene classification” used a rule-based classifier combined with the point-to-plane distance between two epochs to distinguish “changed”, “unchanged”, and “unknown”. Then, changed points were re-classified in a second step into different classes (dormers, roofs, constructions on top roof, cars, and undefined objects) with an accuracy in the range of 80% to 90%. They showed that the quality of the classification results will influence the quality of the change detection.

In the literature, 3D change detection using two ALS epochs is more often investigated in the pre-classification scenario. First change is detected and then it is classified. DSM-based methods were employed in most studies. Murakami et al. [48] operated two multi-temporal ALS data to identify changes in buildings by subtracting the DSMs. Likewise, Vu et al. [50] demonstrated an automatic change detection method to detect damaged buildings after an earthquake in Japan. Pang et al. [49] proposed an object-based analysis method to automatically detect building changes by multi-temporal point cloud data in an 8.5 km^2^ area. Going beyond DSM-based methods, Zhang and Glennie [51] presented a weighted anisotropic iterative closest point (ICP) algorithm, which determined 3D displacements between two point clouds by iteratively minimizing the sum of the squares of the distances. Xu et al. [34] proposed a three-step point-based method for identifying building and tree changes from two LiDAR datasets. The point cloud data were first registered using the ICP algorithm and filtered to extract non-ground points. Then, the non-ground points were stored and indexed in an octree. At last, the buildings and trees that changed were detected by comparing the two LiDAR point clouds and applying the AutoClust algorithm [52].

The aforementioned pre-classification CD studies [34,47,48,49,50,51] have illustrated the possibility of automatic change detection, which can achieve over 80% of accuracy in urban area. Most methods, however, depend on the DSM quality [38] and are concentrated on building changes. Xu et al. [34] has overcome the limitations of previous studies by proposing a method which does not require a DSM and expands the change types to tree cover change in urban area. However, their method has limitations in detecting of the natural growth of trees, which was classified into newly planted trees. Besides, ground points needed to be filtered out in their study. Of course, also ground can change through time and relevant change information should be supplied. In addition to change types, all the methods have the same process: firstly, to separate the “changed” and “unchanged” points, and, afterwards, classify the change types based on the “changed” detection.

Machine learning can be performed supervised, using training data, or unsupervised, with the aim of clustering points with similar features. As the relevant classes in the urban scene are known, we use supervised learning. If only two classes shall be distinguished, Support Vector Machines [53] could be well used. For point cloud classification this is described by [54,55,56,57]. For multiple class problems, Random Forests was suggested [35]. They are efficient and require a moderate amount of training data. Its application to point cloud classification is described, e.g., by [58,59]. Conditional Random Fields (CRF) [60] allow adding context to classification, i.e., considering the relation between neighboring points, and especially were shown to improve the results for classes with fewer points [61,62]. Convolutional neural networks (CNN) were also described for point clouds [63], but they require an extensive set of training data, which is not available in our case. Dictionary learning methods require less training samples but need a long time for processing [64,65,66]. Thus, the method of random forests for point cloud classification was selected.

The new automatic change detection method we suggest targets changes in and between the classes buildings, trees, and ground. A “Changed” and “Unchanged” separation does not need to be performed. Instead, all change types and unchanged types are detected simultaneously based on machine-learning [67].

## 3. Methodology

**Figure 1:**
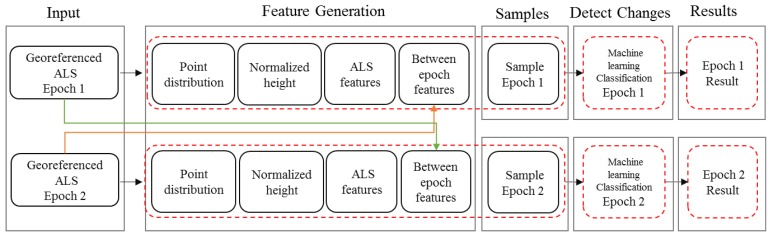
Flowchart of the proposed method.

The proposed method is shown in Figure 1. First, outliers are removed from the data (see Section 3.1). Second, the data of both epochs are merged to compute features of four types: features describing the point distribution, a feature related to height above the terrain, features specific for the multi-target capability of ALS, and features combining both epochs to identify the change (Section 3.2). Training data (Section 3.3) are taken manually, and machine learning (Section 3.4) is applied to compute a model for the classification. Finally, based on the additional attributes of each point, change types are computed (see Figure 1). As shown in Figure 1, each point cloud is classified and investigated for change by an individual machine learning step. All processing is performed in OPALS [68] supported by DTMaster [69] and FugroViewer [70].

### 3.1. Outlier Detection

We assume that the original data are georeferenced already in the same coordinate system and projection. Outlier points, which would cause unpredictable errors in the results, need to be eliminated. Statistics (min, max, mean, and standard deviation) and robust estimators (median and sigMad) of *z* value of the points are computed to set thresholds for outlier detection. Additionally, isolated points are detected and removed. An isolated point is defined as having no neighbors within a certain search radius.

### 3.2. Features

The first set of features describes the point distribution [71]. These features are required for the separability of the classes. Estimation of local planes on a point basis is useful for different tasks (e.g., shaded relief) and surface normals are important geometric properties of a surface. Here, the local tangent plane is estimated by computing the best fitting plane for the ten nearest points. Its normal vector (termed NormalX, NormalY, and NormalZ in the following) and the standard deviation (std.dev.) of the fit are used as additional descriptions of the points (termed NormalSigma). The distribution of the points in the neighborhood, which contain more helpful information, are derived from the structure tensor *T* [72]. Linearity, planarity, and omnivariance are three features obtained from *T*. The linearity feature ($L_{T})$ is used to characterize 3D line objects such as power lines or similar structures. Planarity ($P_{T})$ is a feature which describes the smoothness of the surface and is related to roughness measures. Omnivariance ($O_{T}$) describes volumetric point distributions as they occur for trees. These features are computed using three eigenvalues $\lambda_{1}$ ≥ $\lambda_{2}$ ≥ $\lambda_{3}$ ≥ 0 of the matrix *T* (Equations (1)–(3)).

(1) $$L_{T}=1-\frac{\lambda_{2}}{\lambda_{1}}$$

(2) $$P_{T}=\frac{\lambda_{2}-\lambda_{3}}{\lambda_{1}}$$

(3) $$O_{T}=\sqrt[{3}]{\lambda_{1}\lambda_{2}\lambda_{3}}$$

Different neighborhood definitions are used for the attribute computation of the features EchoRatio, ZRank, and ZRange, which can be derived to provide more information of the points. The EchoRatio is a measure that describes the vertical point distribution and thus the penetrability of the surface [31,73,74]. ZRange represents the maximum height difference between the points in the neighborhood, while ZRank is the rank of the point corresponding to its height in the neighborhood. Thus, the full list of features of the first group is: NormalX, NormalY, NormalZ, NormalSigma, *L_T_*, *P_T_*, *O_T_*, EchoRatio, ZRank, and ZRange.

Secondly, the normalized height is considered as a feature. Mallet et al. [54] have shown that classification of urban areas improves if this feature is considered. However, as we are primarily interested in change detection, the quality of the terrain model is expected to have a lower impact, and thus a simple method [75] is deemed sufficient to compute the DTM, if it is not already available. We use a hierarchic block minimum method (with two iterations). In the first iteration, all the last echo points are selected first. From these points, a raster model is derived by using the “5th lowest” height points in each cell of size 10 m. The height difference of a point and this raster model (nH = *z* (point) − *z* (raster)) is then calculated for each point and all the points in a threshold range above or below the cell elevation are filtered out. For the remaining points the same process (raster creation and nH computation) is repeated using smaller cell size and a smaller nH threshold range in order to obtain the final points for DTM interpolation.

The third set of ALS features exploits the multi-target capability of pulsed LiDAR systems, which can return multiple echoes per emitted laser shot. These echoes are measured directly and the point clouds from ALS not only contain the coordinates (*x*, *y*, *z*) but also further echo info: echo number within the shot, and number of echoes of the shot. Both values are used as features of the point.

**Figure 2:**
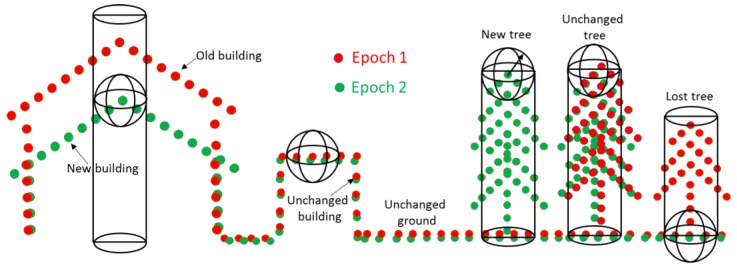
Stability of changed and unchanged objects.

Finally, the fourth set of features are features between epochs. They are computed for each point by considering the distribution of the neighboring points in the other epoch. In Figure 2, the points of the epoch 1 point cloud E1 are investigated for change relative to the point cloud E2. In each point of E1, we search in 3D to find the number $n_{3D}$ of neighboring points of E2 within a sphere of radius R. If this number is zero, there is most likely a change at that point. This is just enough for detecting changes at building and isolated trees, but not for a dense tree area or trees close to buildings. For example, the right red tree in Figure 2 appears in epoch 1 but not in epoch 2. Most of the points in the tree are detected as changed. Nevertheless, this lost tree is close to another unchanged tree, so in the same search radius, some of the lost tree points are still unchanged points because they can find the nearest neighbor of E2 in the unchanged tree. This will be reduced if we consider also a 2D search around the same point to find the number $n_{2D}$ within a cylinder of radius R. Finally, the ratio of these point numbers in percent is called “stability” (Equation (4)). This is identical to EchoRatio, with the difference that the point of evaluation if from a different point set than the points counted in the 2D and 3D neighborhood.

(4) $$Stability=\;\frac{n_{3D}\times 100}{n_{2D}}$$

where $n_{3D}$ is the number of points found in a fixed search distance (e.g., 1 m) measured in 3D (i.e., search sphere); and $n_{2D}$ is number of points found in the same distance measured in 2D (i.e., vertical search cylinder with infinite height).

### 3.3. Training Sample

The training samples provide information to the learning system. The supervised learning algorithm analyzes the training data and produces an inferred function, which can be used for mapping the remaining data. The training sample quality directly influences the classification results, as label noise, i.e., a wrong class decision on a training point, influences the learned function for the classification. All the changed types are taken thoroughly by manual selection. In this study, the change samples follow the rules shown in Table 2. It is not necessary to foresee changes in all classes and in this experiment the class water was not investigated for change.

### 3.4. Change Types Classification

As a state-of-the-art machine learning algorithm, random forest [35] is used to classify the urban area because of its advantages. It does not overfit, runs fast and efficiently for a large dataset such as LiDAR [58] and it requires a moderate amount of training data. This method is useful for automatic classification of urban objects. All the sample points contain the four sets of features (mentioned in Section 3.2). Random forest selects randomly features for subsets of the sample points to train several decision trees. Each randomly created decision tree is used to predict the class of a new (unseen) point based on its features and stores this outcome. The highest voted predicted outcome is considered the final classification for each point. The final classification model is then applied to the rest of the point cloud to generate the final change detection classification results.

## 4. Study Site

**Figure 3:**
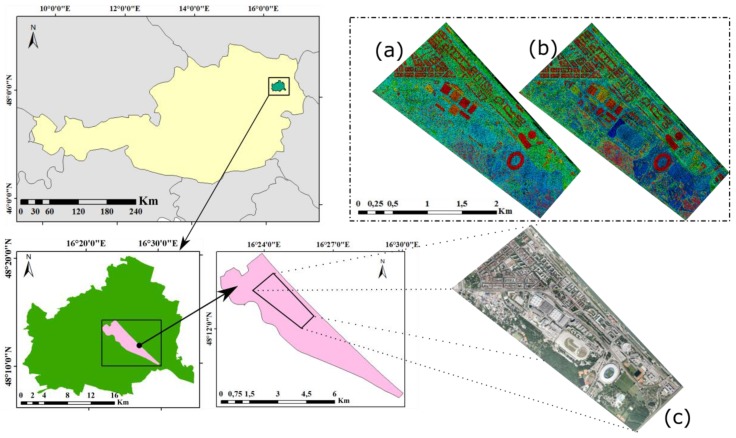
Experiment area: (**a**) LiDAR data for 2007; (**b**) LiDAR data for 2015; and (**c**) Google Earth image of experiment area.

The Leopoldstadt District, in Vienna, Austria, is taken as the study area. The experimental region (Figure 3), covering an area of about 3 km × 1.5 km, is generally flat. It contains complex objects, containing several old-fashioned and modern high-rise buildings, a suburban area with mainly single-dwellings, an open-wide area (including a stadium), water, overpasses, an amusement park, and a variety of other objects. Since 2005, this area has been one of the most dynamic areas with respect to changes in land use in Vienna. Old buildings have been rebuilt into new buildings or open ground, new buildings are constructed from bare ground and cut trees, new trees are planted suitable for the landscape, and a new road and a stadium construction was built. All these led to changes in buildings, vegetation, and ground in this area.

Two sets of LiDAR data are available, which were obtained in 2007 (from 7 December 2006 to 5 February 2007) and 2015 (9–24 November 2015). These data have average point densities of 12 points/m^2^ measured with a Riegl LMS_Q560 (Riegl, Horn, Austria) and 16 points/m^2^ measured with a Riegl LMS_Q680i, respectively. As the datasets are already registered well, no extra steps for registration were required. Ortho-photos from the time of flight were not available, and thus Google earth images of the respective years were used to support interpretation of the main objects. This was necessary for taking training samples for machine learning, as well as a manual classification of the point cloud for the accuracy assessment process at the end.

## 5. Results and Discussion

The highlight of our change detection method is the combination of the steps of change detection and change classification in one single step based on the stability value combined with the other attributes in order to classify all objects into different classes, comprising: unchanged points (ground, building, and tree), and changed points (new building, lost buildings, new tree, lost tree, ground changed in height, and ground changed into other objects). The final results are then evaluated using a point-based accuracy evaluation by comparing the automatic change detection classification results with the manual change detection classification.

### 5.1. Stability Feature

Stability (Equation (4)) is a feature which is used to detect change points in this paper. A good estimate for the selection of a suitable search radius is the double of the average point spacing found in the study area. This guarantees a representative number of neighbors, while avoiding too large neighborhoods, which would cause expanded transition zones at the border of two objects with different surface structure [73]. A search radius of 1.0 m is chosen in this paper. In flat open terrain this will result in around 38 neighboring points for 2007 and around 50 points for 2015. If no points of E2 are found by 3D search, the value of stability is 0%. That point is then a changed point. In the case of unchanged points, buildings and ground have low transparency, the number of 3D and 2D neighbors of E2 should be approximately the same, so resulting in a high stability (100%). In contrast, vegetation is transparent to LiDAR shots (to some degree) and thus the laser point density on and within vegetation depends on the density of the branches, the twigs and the leaves. The latter even depends on the vegetation type and the time of year. Consequently, one has to expect lower stability values at vegetation objects.

**Figure 4:**
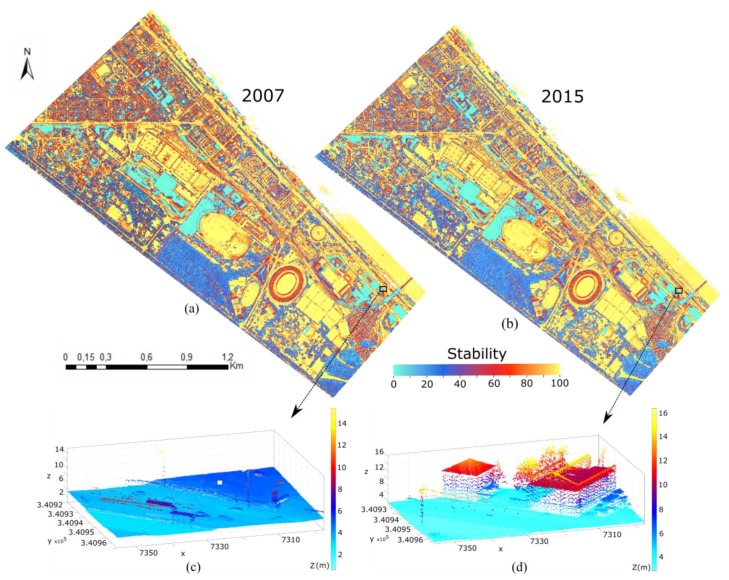

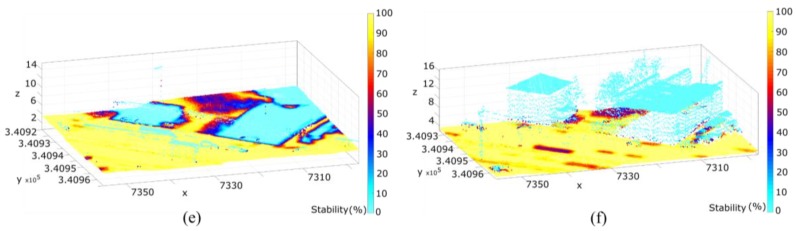
Stability feature for change detection: (**a**) stability shown for 2007 as color value, reaching from 0 (cyan) to 100% (yellow); (**b**) stability shown for 2015; (**c**) point cloud of 2007 shown as height value in an specific area; (**d**) point cloud of 2015 shown as height value in an specific area; (**e**) stability value range in specific area of 2007; and (**f**) stability value range in specific area of 2015.

Figure 4 presents a rasterized image of the stability value for each of both datasets. From these images, it can be seen that the changed and unchanged regions are detected. Figure 4a,b shows the stability value ranges from 0 to 100% for the whole study area in epoch 2007 and 2015, respectively. To be perfectly clear in detail, a small specific area is zoomed in and indicated in height value (Figure 4c,d). Figure 4e,f indicates the stability value of this area. Changed buildings and grounds obtain a very low value (approx. 0%).

### 5.2. Sampling Training Data and Classification

**Figure 5:**
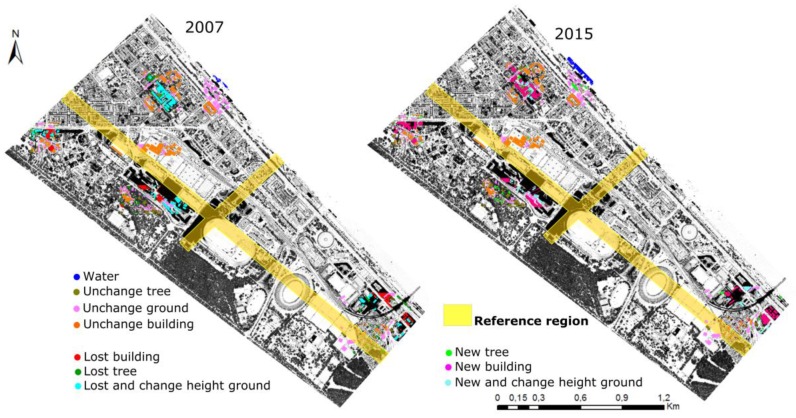
Sample points distribution in both datasets. The area that was used as reference for the overall evaluation, i.e., the accuracy assessment of the classification results, is shown in yellow.

Because of the large area, the sample regions were taken from six small regions, where the changes took place actively. The samples of the unchanged objects (i.e., unchanged grounds, unchanged buildings, and unchanged trees) and grounds changed in height were taken simultaneously for both datasets. “Lost tree” and “Lost buildings” samples are only taken in 2007, whereas “New trees” and “New buildings” are only taken in 2015. The training samples were taken in DTMaster software. Table 3 sums up the number of sample points in each class. Seventy percent of the training data is used for learning, whereas the remaining 30% is used for evaluating of the learned random forest classification model (but not for the overall evaluation—different reference data are used for that; see Figure 5). Figure 5 displays the sample distribution in both datasets over the whole experiment area. The data for overall evaluation are overlaid with a yellow cross (see Section 5.4).

**Figure 6:**
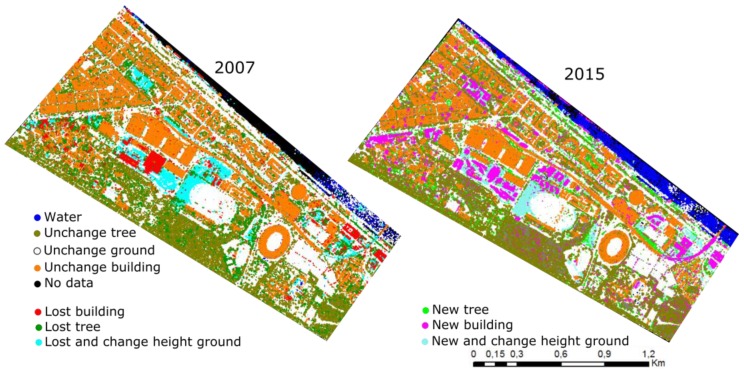
Change detection classification results of 2007 and 2015 datasets.

After taking the sample points, they are used for training and creating the classification model for each dataset (one model for 2007 dataset, and one model for 2015 dataset). The models were then applied to the whole area to obtain the final change detection classification results in both datasets (Figure 6). The total number of processed points are 97,809,515 and 117,734,603 points in 2007 and 2015 datasets, respectively. The time for creating the models from the samples and applying the models to the total points in two datasets took 1:41 h for 2007 and 2:49 h for 2015 on a single PC with a Windows 7 Enterprise system (AMD FX ™-6100 Six-Core Processor, 16G RAM) (Singer Computer, Vienna, Austria). 

**Figure 7:**
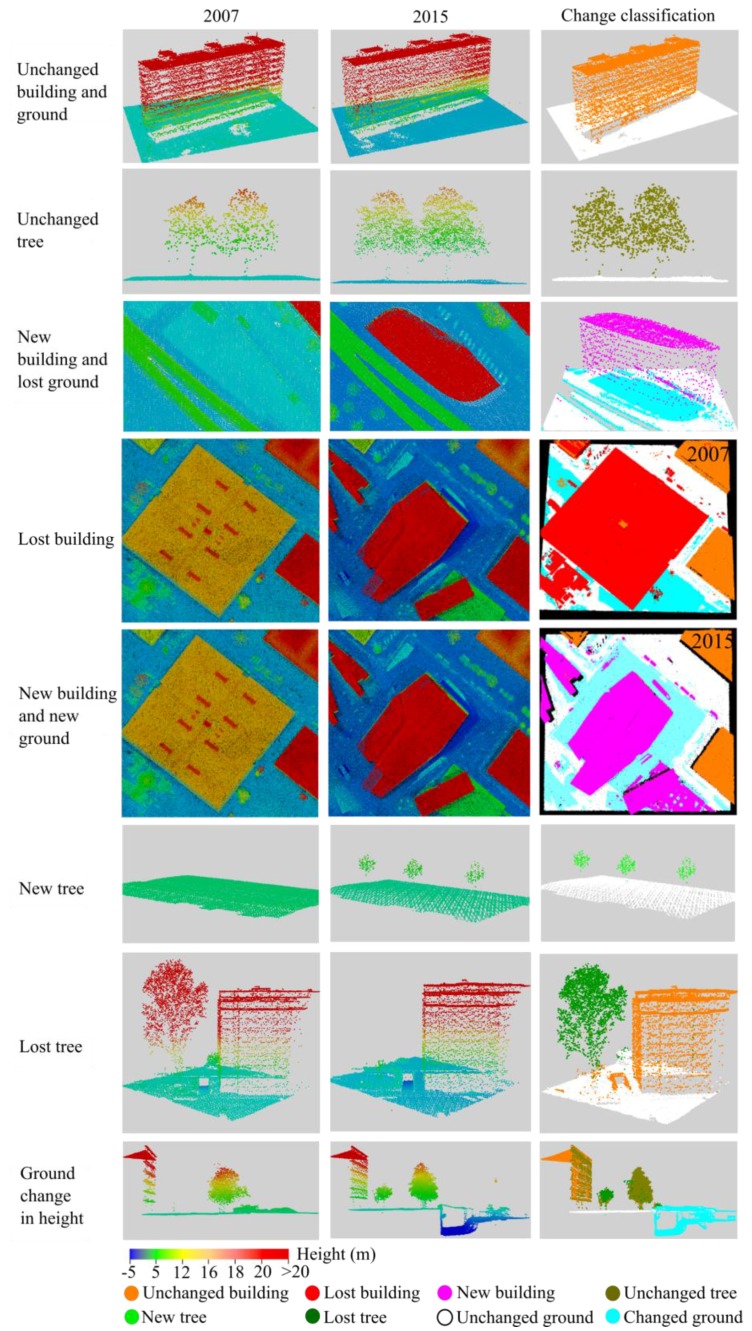
Example of change types of classification results. The first two columns show point clouds from 2007 and 2015, respectively, with color indicating height (legend below images). In the right column both point clouds, 2007 and 2015, are shown with their class label, with exception of the building in row 4 and 5, where the point clouds are shown separately for each year.

As can be seen in Figure 6, the results of the proposed method are satisfactory, thus indicating that the method is effective for the complex urban area. All the unchanged and changed objects were detected simultaneously. A visual comparison of both results in Figure 6 shows that the changes in 2007 and 2015 correspond nicely to one another; i.e., where there is a change in 2007 (with respect to 2015) change in 2015 (with respect to 2007) also appears. The same holds true for the unchanged objects. Figure 7 shows in detail the change type classification results. The first and the second column show the objects in the data 2007 and 2015. Points are colored by elevation blue to red. The third column shows the change detection and classification results of the change types.

### 5.3. Impact of Using the Raw Point Cloud

**Figure 8:**
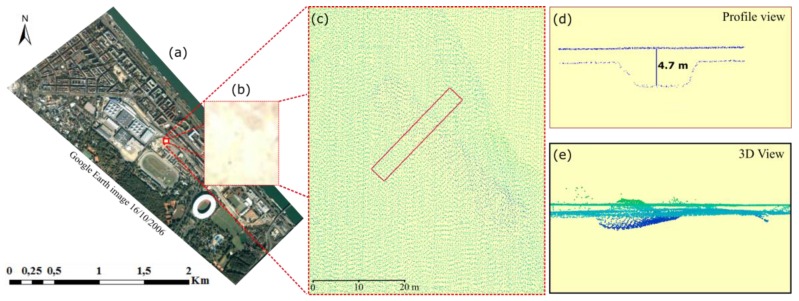
Open ground in the point cloud of 2007. Two contradicting layers of ground are shown due to construction activity within the duration of the 2007 campaign. (**a**) The Google earth image shows the location of the construction area; (**b**) orthophoto showing the selected area; (**c**) ground view of the point cloud indicates the position of the profile shown in sub-figure (**d**); and (**e**) a 3D view of the multilayer point cloud with the difference in height reaching 4.7 m.

**Figure 9:**
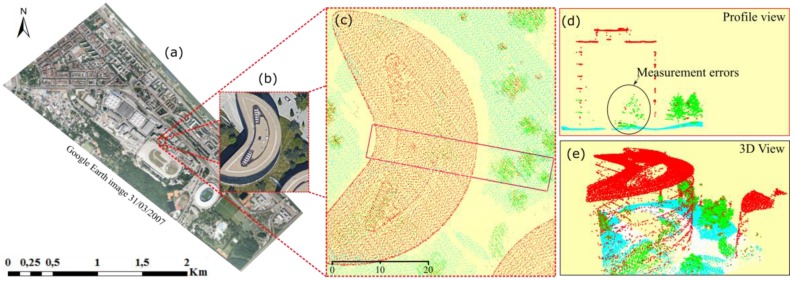
Erroneously measured points inside a mirroring building with a glass façade in the 2015 dataset. (**a**) The Google earth image located the position of the building; (**b**) orthophoto showing the selected area; (**c**) the ground view of the point cloud; (**d**) the profile view displays the erroneously measured point inside the building; and (**e**) 3D view of the point cloud.

The data used are several years apart. Such a time difference is suitable for new constructions. Vegetation objects may change considerably in the long period because of their natural growth. Additionally, each dataset itself contained certain problems, apart from outliers removed beforehand. Because of the duration of the measurement campaign, in the active change areas also changes within the overlap of 2007 LiDAR strips were found. It contained different objects (e.g., difference in ground) at the same position. Figure 8 shows a ground height difference of 4.7 m at the same location. This violates the underlying assumption of a stable object within one epoch and leads to a wrong classification in the ground of the 2007 dataset. In the 2015 dataset, because of a building wall material acting as a mirror, trees and grounds are wrongly recorded inside the buildings (Figure 9). Those wrong measurements could also not be discovered as noise in the outlier removal step. These problems were identified when collecting the reference data by the manual operator. Although all wrong points are removed as high point in the accuracy assessment step (see below), they have an impact on the final results because they influence the real points in the step of calculating attributes which are used for the classification. 

### 5.4. Accuracy Evaluation

To evaluate the effectiveness of the proposed methods for detecting changes in urban areas, a comparative analysis between change detection results and the reference data was conducted. Because no photos were acquired which were collected simultaneously with the ALS data, the reference data were obtained by visual interpretation in 3D and manual classification. Reference point collection was conducted based on the rules:

- Unchanged buildings: The same geometric building in two epochs or buildings which have changes in roof but lower than 1 m (i.e., paying tribute to the chosen search radius).
- Lost buildings: Buildings are available in the older data but not in the later data.
- New buildings: Buildings are available in the later data but not in the older data.
- Unchanged ground: The height of the ground did not change more than 0.5 m.
- Changed ground: The ground has changed in height, changed to other types of land use (i.e., new buildings), or new ground.
- Unchanged trees: Trees at the same position.
- Lost trees: Trees that were cut.
- New trees: Newly planted trees.
- High points: Cars, fences (wooden, concrete, metal, and small bushes), wires, ships on the water, etc.

This manual classification approach is a tough task and time-consuming. However, this approach is more advantageous than using orthophotos in the case of comparing the change in height of ground, which is difficult when using 2D orthophotos. The selected region for doing manual classification, which is shown in Figure 5, was cut out from the datasets. The criteria to choose this area were: (1) select an area where all types of changes occur; (2) avoid the training samples as much as possible to ensure the objectivity of the accuracy assessment; and (3) investigate the entire study region, also for objectivity. The total reference area is about 33.7 ha out of the whole area 376.7 ha (approximately 9%). The time spent for manual classification of this area was about 150 working hours.

To focus on the above-mentioned changed objects only, the “high points” are manually classified but not investigated for change. They contain primarily objects, which are dynamic within a day, and objects for which the sampling is rather poor (thin objects). Those high points also removed from the accuracy evaluation. In addition, the class water is not represented in the confusion matrix. High points and water together add up to 3% of the ground truth data. The evaluated points are grouped into classes according to object change: unchanged ground (UG), changed ground (CG), unchanged building (UB), lost building (LB), new building (NB), unchanged tree (UT), lost tree (LT), and new tree (NT). The confusion matrix documenting the accuracy are shown in Table 4. Both datasets achieved a high overall accuracy of about 91% and 92% in 2007 and 2015, respectively.

From Table 4 it can be seen, that five out of six classes show over 80% correctness in the 2007 dataset. Only the class UT reached 70.7% of correctness because of misclassification as unchanged building (1.1%) and lost tree (0.5%).

There are some specific problems, most relating to special objects in the dataset. Unchanged building points are misclassified as unchanged tree in the case of complex building roofs, especially at the edge of the buildings, and the stadium frame dome where the distribution of points is the same as the tree point distribution. 

**Figure 10:**
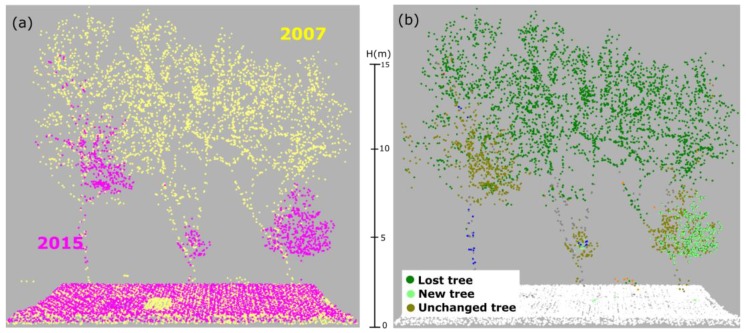
New trees planted at the same location as old lost trees: (**a**) point clouds of 2007 and 2015; and (**b**) change detection and classification results.

In the confusion matrix of the 2015 dataset (Table 4), the most striking class is NT (new trees), for which correctness and completeness reach only 58% and 56.5%, respectively. Here, about 39% (1.1/2.8) of the points that in reality are NT were wrongly classified as UT (unchanged trees). The reason for this low completeness can be explained by two reasons. Firstly, some old trees were cut and at the very same position new trees were planted (see Figure 10). Consequently, in the change detection result, these tree points are classified as unchanged trees. Some new small trees grow near big cut trees (Figure 10) and are also mis-detected.

**Figure 11:**
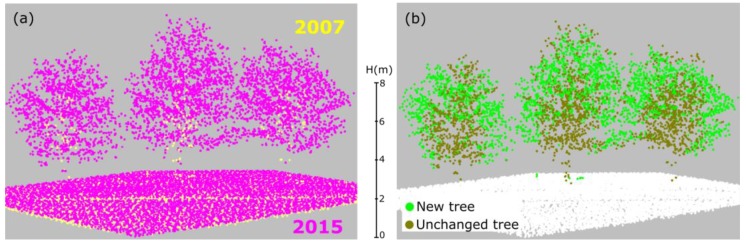
New planted trees at the same location with lost tree: (**a**) point cloud in 2007 and 2015; and (**b**) change detection and classification results).

Because of roughly eight years apart, trees grow up significantly for the small trees and the grown trees have changed their shape (e.g., branch cut and new branch). Consequently, those growing points are classified into new trees, but in reality, they are parts of the same tree. This leads to a low completeness in new tree points of the 2015 ALS data (Figure 11). 

A visual analysis of the entire change detection result suggests that the following classes can be determined with high reliability: unchanged ground, changed ground, new building, lost building, unchanged building, lost tree. However, this analysis also revealed that the change detection for growing trees constitutes a big challenge, as some unchanged tree points were classified as new buildings. This originates in a very dense canopy cover during the 2015 data acquisition resulting in a planar distribution of points and therefore features which are more similar to building points. This can be seen in the forested areas of 2015 on the southwestern border of the dataset (see Figure 6). By selecting points of the respective classes, we estimated that about 1.5% of unchanged tree points are wrongly classified as new buildings.

### 5.5. Discussion

Thus far, most studies focused on change detection of buildings only and they achieved very high accuracy. A very recent research which is closest to our study is the one by Xu et al. [34]. Their overall accuracy for change detection of buildings and trees reached 94.8% and 83.8%. However, their method to access the accuracy is different from ours. Their reference data was obtained by visual interpretation of the aerial images and airborne LiDAR data from two dates counting changed objects (not points). Then, their experimental results and the reference data were put together. The correctness and completeness of the buildings are determined visually based on the count and the area respectively. Our method does not only evaluate more objects compared to their method, but also our comparison is based on the classification of corresponding points, not on object count and area. Thus, the accuracy values cannot be compared directly.

The classification results in Figure 6 and the evaluation outcomes in Table 4 demonstrate the good performance of the proposed method in urban areas. This method exploits the ability of extending machine learning classification to perform classification and change detection in one step. In addition to combining change detection steps, the proposed method also is flexible in feature selection as well as in the data used. Firstly, in feature selection, for 3D point clouds numerous features can be used for a machine learning classifier. Weinmann et al. [71] mentioned numerous 2D and 3D features. In our study, we just used some of these features. However, depending on the classification task, the selection of features may be extended or compacted (e.g., using color in point clouds from image matching). In addition, the change detection feature used in this study is “Stability” to detect the changes between two epochs. However, other features, such as difference in roughness value of the local point to the nearest point of the other epoch, surface distance between one point in one epoch to the surface of the nearest points in the other epoch (compare tangent plane ICP correspondence [76]) etc., can be used as alternative sources of change information. To investigate this, new features were investigated: (1) distance to nearest point in the other epoch, (2) difference in roughness of current point and the nearest point in the other epoch. With these features new models were learned and the classification performed for the entire dataset. Using only the distance feature, the overall quality 91% decreases slightly to 89%, using only the difference in roughness it drops to 73%. Using all three features as markers of change, the overall accuracy increases in the order of 0.1% to 91% and 92% for 2007 and 2015, respectively. Secondly, the proposed method has potential for combining different data sources for change detection. With the flexibility in feature selection, our method allows doing change detection and classification for different data depending on the features given to identify the classes. Image matching point clouds recently became one of the important sources used in urban classification, also exploiting the provided color information. This data can be applied in further studies for change detection in urban area where the changes in buildings, trees, and grounds occur frequently.

**Figure 12:**
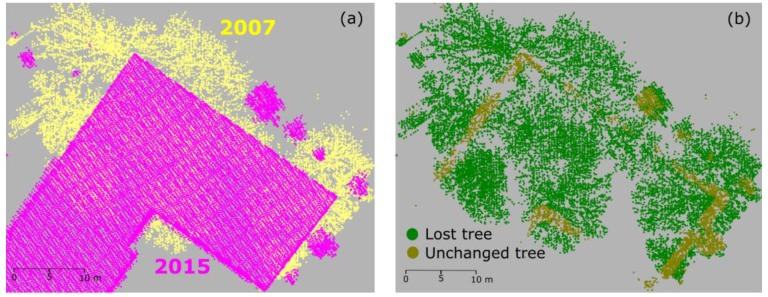
Example of misclassification in the case of object with adjacent old and new data: (**a**) data in 2007 and 2015; and (**b**) misclassification results in 2007. In the intersection of the objects, points are misclassified as unchanged trees.

Although the proposed method obtained a satisfying change detection classification result in one step compared to other studies, there remain some limitations. Firstly, the results of classification strongly depend on the training samples. Especially for a complex urban area, it is required to consider various types of objects. Thus, to select the samples of each class required careful observation and selection. Secondly, in the case of changes where old and new points are too close to each other, the method did not work well. For example, cut trees and a new building are shown in Figure 12. Post classification methods (e.g., label smoothing [77]) may support improvement of the results. Thirdly, as mentioned above, growing tree points are mis-detected as new trees. It is difficult to separate this class (growing tree points) from the new tree class. A solution may require object detection, i.e., single tree detection in this case. Parameters of individual trees could then be compared.

Finally, we compared our method to a traditional two-step classification approach, i.e., detect the change and then classify the changes. Two DSM models of 2007 and 2015 are derived with a cell size of 1 m. The DSM difference of 1 m is chosen to separate changed and persistent objects. The first three sets of features (i.e., point distribution, height above the terrain, ALS features) are rasterized with the same cell size of 1 m. Those rasterized images are masked into a changed and an unchanged region based on the DSM masks. The training samples are rasterized and divided into changed and unchanged samples for each epoch 2007 and 2015. Based on those samples, the classification is performed. Finally, combining the masks and the classification result, the change detection classification is performed for 2007 and for 2015. This traditional raster-based approach is easy to process and less time is required for processing compared to our point-based method. However, the final results depend on the first change detection step. DSM-based change detection is useful for buildings, but not for trees. Tree growth can be higher than 3 m, given the eight years apart. Therefore, if the DSM difference is 1 m, unchanged trees are classified into new trees. Increasing the DSM difference, the change in ground and small buildings are lost. For this reason, the overall accuracy of this method is only 78% for both the 2007 and the 2015 datasets. Furthermore, the raster-based method does not feature the 3D content anymore, which is available in the point-based method.

## 6. Conclusions

This paper has presented a fusion of automatic classification and change detection using a supervised point-based machine learning approach to infer changes in the objects building and tree, as well as changes of the ground. The main contribution of the proposed method can be summarized as: (1) the proposed method establishes a complete set of processes to detect and classify changes in buildings, trees and ground; and (2) not only are changes detected, but they are also simultaneously classified, which had not been done before, especially for the major classes ground, building, and tree in one step. The combination of the “Stability” feature with other attributes plays an important role for the automatic change detection and classification of different types of urban objects. The overall accuracy of the final classification of each change type of the 2007 dataset and 2015 dataset reached 90.93% and 92.04%, respectively. Therefore, the proposed method can be used as an alternative method for detecting changes in urban areas in high resolution point clouds.

## Figures and Tables

**Table 1:** List of proposed change detection approaches.

Authors	Year	Data Used			CD Approach	CD Classes
		ALS	Image	Maps		
Matikainen et al. [36]	2004	X	X	X	Post-classification	Building
Matikainen et al. [37]	2010	X	X	X	Post-classification	Building
Stal et al. [38]	2013	X	X		Post-classification	Building
Malpica et al. [39]	2013	X	X		Post-classification	Building
Matikainen et al. [40]	2016	X	X	X	Post-classification	Building
Matikainen et al. [41]	2017	X	X	X	Post-classification	Building, roads
Vosselman et al. [42]	2004	X		X	Post-classification	Building
Tang et al. [43]	2015	X		X	Post-classification	Building
Awrangjeb et al. [44]	2015	X		X	Post-classification	Building
Choi et al. [45]	2009	X			Post-classification	Ground, vegetation, building
Xu et al. [46]	2015b	X			Post-classification	Building
Teo et al. [47]	2013	X			Post-classification/DSM-based	Building
Murakami et al. [48]	1999	X			Pre-classification/DSM-based	Building
Pang et al. [49]	2014	X			Pre-classification/DSM-based	Building
Vu et al. [50]	2004	X			Pre-classification/DSM-based	Building
Zhang et al. [51]	2014	X			Pre-classification	Ground
Xu et al. [34,46]	2015a	X			Pre-classification	Building, tree

**Table 2:** Rules of taking sample for machine learning classification.

Change Objects	Change Types	Description
Buildings	Unchanged high-building	The same high-building is in both epochs
	Unchanged low-building	The same low-building is in both epochs
	New high-building	New building with height >15 m
	Lost high-building	Lost building with height >15 m
	New low-building	New building with height ≤15 m
	Lost low-building	Lost building with height ≤15 m
	New walls	Walls in new building
	Lost walls	Walls in lost building
	Unchanged walls	Walls in unchanged building
Trees	New tree	New planted tree
	Lost tree	Cut tree
	Unchanged trees	The same tree in both periods
Ground	Unchanged ground	The same ground or absolute height differences ≤0.5 m
	Change in height	Ground has absolute height differences >0.5 m
	New ground	Buildings changed to grounds
	Lost ground	Ground changed to buildings
Water	Water	Water points

**Table 3:** Sample points of different change types in 2007 and 2015 datasets.

Change Types	Sample Points 2007	Sample Points 2015
Unchanged grounds	698,323	639,465
Unchanged low buildings	181,022	169,015
Unchanged high buildings	443,891	463,812
Unchanged walls	44,504	43,796
Lost walls	9341	-
New walls	-	62,795
New high building	-	479,565
Lost high building	65,653	-
New low building	-	53,219
Lost low building	189,327	-
Lost tree	193,035	-
New tree	-	138,402
Unchanged trees	184,781	515,326
Ground change in height	113,662	85,766
New ground	-	51,919
Lost ground	373,161	-
Water	2400	40,703

**Table 4:** Confusion matrix of the classification result for the 2007 and 2015 datasets. The rows correspond to the reference classification, the columns to the automatic classification. EOO, Error of Omission; Comp, Completeness; EOC, Error of Commission; Corr, Correctness; UG, unchanged ground; CG, changed ground; UB, unchanged building; LB, lost building; NB, new building; UT, unchanged tree; LT, lost tree; NT, new tree.

**2007**	**UG**	**CG**	**UB**	**LB**	**UT**	**LT**	**Ref Sum**	**EOO**	**Comp**
Ref_UG	53.8	1.8	0.1	0	0.1	0	55.8	3.6	96.4
Ref_CG	3.6	10.1	0	0.1	0	0	13.8	26.7	73.3
Ref_UB	0.1	0	16.7	0.4	1.1	0.1	18.2	8.7	91.3
Ref_LB	0	0	0.2	2.9	0	0.1	3.1	8.6	91.4
Ref_UT	0	0	0.4	0	4.1	0.4	4.9	16.1	83.9
Ref_LT	0	0	0.1	0.1	0.5	3.4	4.1	18.2	81.8
Sum	57.5	12	17.4	3.5	5.8	3.9	100	0	100
EOC	6.4	15.6	4.2	17.6	29.3	13.2	0	0	100
Corr	93.6	84.4	95.8	82.4	70.7	86.8	100	100	0
Overall Accuracy: 90.93									
Total number of points: 8,542,450									
**2015**	**UG**	**CG**	**UB**	**NB**	**UT**	**NT**	**Ref_sum**	**EOO**	**Comp**
Ref_UG	48.3	0.5	0.1	0	0	0	48.9	1.3	98.7
Ref_CG	0.9	10	0.1	0.1	0	0	11.0	9.1	90.9
Ref_UB	0	0	16.5	0.2	0.9	0	17.7	6.9	93.1
Ref_NB	0	0.2	0.1	4.6	0.1	0	5.0	8.4	91.6
Ref_UT	0	0	0.3	0.2	11.1	1.1	12.8	12.9	87.1
Ref_NT	0	0	0.1	0.1	1.1	1.6	2.8	43.5	56.5
Sum	49.2	10.8	17.1	5.3	13.2	2.7	98.2	0	100
EOC	1.8	6.9	3.6	12.4	15.9	42	0	0	100
Corr	98.2	93.1	96.4	87.6	84.1	58.0	100	100	0
Overall Accuracy: 92.05									
Total number of points: 8,636,900									
